# Prevalence and Distribution of *Listeria monocytogenes* in Three Commercial Tree Fruit Packinghouses

**DOI:** 10.3389/fmicb.2021.652708

**Published:** 2021-06-10

**Authors:** Tobin Simonetti, Kari Peter, Yi Chen, Qing Jin, Guodong Zhang, Luke F. LaBorde, Dumitru Macarisin

**Affiliations:** ^1^Department of Food Science, The Pennsylvania State University, University Park, PA, United States; ^2^Department of Plant Pathology and Environmental Microbiology, The Pennsylvania State University, University Park, PA, United States; ^3^Center for Food Safety and Applied Nutrition, Food and Drug Administration, College Park, MD, United States

**Keywords:** *Listeria monocytogenes*, contamination, tree fruit, packinghouse, postharvest

## Abstract

A 2-year longitudinal study of three tree fruit packinghouses was conducted to determine the prevalence and distribution of *Listeria monocytogenes*. Samples were collected from 40 standardized non-food-contact surface locations six different times over two 11-month production seasons. Of the 1,437 samples collected, the overall prevalence of *L. monocytogenes* over the course of the study was 17.5%. Overall prevalence did not differ significantly (*p* > 0.05) between each year. However, values varied significantly (*p* ≤ 0.05) within each production season following packing activity levels; increasing in the fall, peaking in early winter, and then decreasing through spring. *L. monocytogenes* was most often found in the packing line areas, where moisture and fruit debris were commonly observed and less often in dry cold storage and packaging areas. Persistent contamination was attributed to the inability of water drainage systems to prevent moisture accumulation on floors and equipment during peak production times and uncontrolled employee and equipment traffic throughout the facility. This is the first multiyear longitudinal surveillance study to compare *L. monocytogenes* prevalence at standardized sample sites common to multiple tree fruit packinghouses. Recommendations based on our results will help packinghouse operators to identify critical areas for inclusion in their *L. monocytogenes* environmental monitoring programs.

## Introduction

Transmission of *Listeria monocytogenes* to humans is responsible for listeriosis, a relatively rare but serious illness that in the United States accounts for only 0.02% of all the cases of foodborne disease yet is responsible for 19% of food-related deaths (Scallan et al., 2011; Donovan, 2015). Outbreaks of listeriosis are usually limited to susceptible populations that include the very young, the elderly, and those with suppressed immune systems (Swaminathan and Gerner-Smidt, 2007). The primary reservoir for *L. monocytogenes* is animals, including cattle, sheep, and goats, and it is widely found in produce growing environments (Strawn et al., 2013a; Chapin et al., 2014). This psychrotrophic pathogen is of particular concern in food processing facilities that are continuously moist and cool, and where nutrients (food debris) are abundant (Carpentier and Cerf, 2011; Ferreira et al., 2014; Murugesan et al., 2015; Buchanan et al., 2017). Once *L. monocytogenes* enters a facility and adapts to the food and the environment, it is difficult to eliminate without implementation of a rigorous *Listeria* control program (Lappi et al., 2004b; Murugesan et al., 2015; UFPA, 2018).

Outbreaks and recalls associated with *L. monocytogenes* have been historically associated with smoked and raw fish (Hoffman et al., 2003; Chen et al., 2016b), poultry (Lawrence and Gilmour, 1994; Rothrock et al., 2019), ready-to-eat deli meat (Sauders et al., 2009; Williams et al., 2011; Simmons et al., 2014), and raw milk or dairy products (Gombas et al., 2003; Ho et al., 2007; Kim et al., 2018). However, an increasing number have been linked to fresh produce (Garner and Kathariou, 2016; Buchanan et al., 2017; Zhu et al., 2017). Post-harvest *L. monocytogenes* contamination within the packing or processing environment has been linked to several recalls and outbreaks of produce including cantaloupe (FDA, 2011), onions (CDPH, 2012; FDA, 2016c) diced celery (Gaul et al., 2013), sprouts (CDC, 2015a), avocados (FDA, 2019b), and leafy green salads (FDA, 2016b; Self et al., 2019).

Tree fruit has long been thought to present fewer food safety risks compared to fruits and vegetables that grow closer to the ground and that are therefore more likely to come into contact with soil that can be a significant source of pathogens (Strawn et al., 2013b). However, in the last several years, the number of recalls and outbreaks traced to orchard grown fruit contaminated with pathogens has increased, suggesting that fresh tree fruits are at risk for exposing consumers to *L. monocytogenes*. In 2014, a California packing company voluntarily recalled whole peaches, nectarines, plums, and pluots after internal testing revealed that some lots were contaminated with *L. monocytogenes* (Jackson et al., 2015). Further testing revealed that over half of the fruit contained detectable levels of *L. monocytogenes* (Chen et al., 2016a). Also, in 2014, Del Monte Fresh announced a voluntary recall of fruit mixes containing Gala apples because *Listeria* contamination was found in the supplying packinghouse (FDA, 2014). The same company issued another recall in 2015 after *L. monocytogenes* was found on whole Granny Smith apples used to prepare sliced products (FDA, 2015). A multistate outbreak of listeriosis was attributed to the consumption of prepackaged caramel apples, which caused 35 hospitalizations and seven deaths (CDC, 2015b). Although *L. monocytogenes* does not grow on the surface of apples during storage (Sheng et al., 2017; Macarisin et al., 2019), experimental studies have demonstrated that punctured apples coated in nutrient rich caramel can create conditions favorable for the growth of the pathogen (Glass et al., 2003; Salazar et al., 2016). Again in 2015, sliced apples and mixed fruit products produced in Canada were recalled after one case of listeriosis occurred, and *L. monocytogenes* was found in some of the tested products (CFIA, 2015). In 2016, multiple salad products containing sliced apples were recalled by a Texas company after *L. monocytogenes* was detected in random product samples (FDA, 2016a). In 2017, A Michigan fresh-cut apple facility recalled apple slices (FDA, 2017a) after routine testing at the packinghouse supplier indicated *L. monocytogenes* contamination (FDA, 2017b). Caramel apples epidemiologically linked to the Michigan apples were recalled as a precautionary measure, and it was later found that the strains of *L. monocytogenes* found in the packing facility were closely related to clinical isolates taken from hospitalized patients (Marus et al., 2019). In 2019, Chilean grown peaches, nectarines, and plums distributed in several United States states were recalled after *L. monocytogenes* was found on some fruits during routine packinghouse testing (FDA, 2019a). In 2020, California grown whole peaches shipped to several United States states and multiple foreign countries were implicated in an outbreak of salmonellosis that afflicted 101 persons across 17 states (FDA, 2020). Several of these recalls and outbreaks have triggered retail stores and processors who purchased the implicated fruit to issue their own market withdrawals.

The 2011 Food Safety Modernization Act (FSMA) [P.L. 111–353] requires food establishments that pack, hold, or distribute food to conduct risk-based assessments of their operations to minimize the chance for consumers to become ill or injured from the foods they eat. Under the Current Good Manufacturing Practice, Hazard Analysis, and Risk-Based Preventive Controls for Human Food Rule (Preventive Controls Rule; Federal Register, 2015) FDA requires facilities, where ready-to-eat food is exposed to the environment prior to packaging and the packaged food is not subjected to a microbiological control measure, to monitor the presence of *Listeria*. Guidance on developing environmental monitoring programs (EMP) and corrective actions to take when samples are positive for *L. monocytogenes* have been widely adopted (FDA, 2017c; Zoellner et al., 2018).

Several recent outbreaks and recalls have demonstrated that strategies to prevent *L. monocytogenes* contamination in RTE facilities, particularly in the fresh produce industry, have not been entirely effective. This could be attributed in part to a limited understanding of the occurrence and distribution of this pathogen in packinghouses. *L. monocytogenes* testing methods that are recommended for environmental monitoring programs have been validated under laboratory conditions. However, their performance in recovering stressed cells can vary (Sheth et al., 2018). Furthermore, EMP results are not usually available publicly and thus barely contribute to bridging the knowledge gap on *L. monocytogenes* occurrence and distribution in fresh produce packing and processing facilities.

Given the recent history of *L. monocytogenes* contamination of tree fruit, there is a need to determine the prevalence, distribution, and routes of contamination of this pathogen in packinghouses. We employed FDA testing methods, recently demonstrated to be efficient in recovering and detecting stressed *L. monocytogenes* cells from environmental samples (Sheth et al., 2018), to generate multiyear comprehensive reference data on *L. monocytogenes* incidence and prevalence in three tree fruit packing facilities.

## Materials and Methods

### Study Locations

Each of the three tree fruit packinghouses in this study were independently owned and operated within a 165-Km^2^ area. Tree fruit packed at each location were primarily sourced from orchards located no farther than 640 km from the packinghouse.

**Figure 1:**
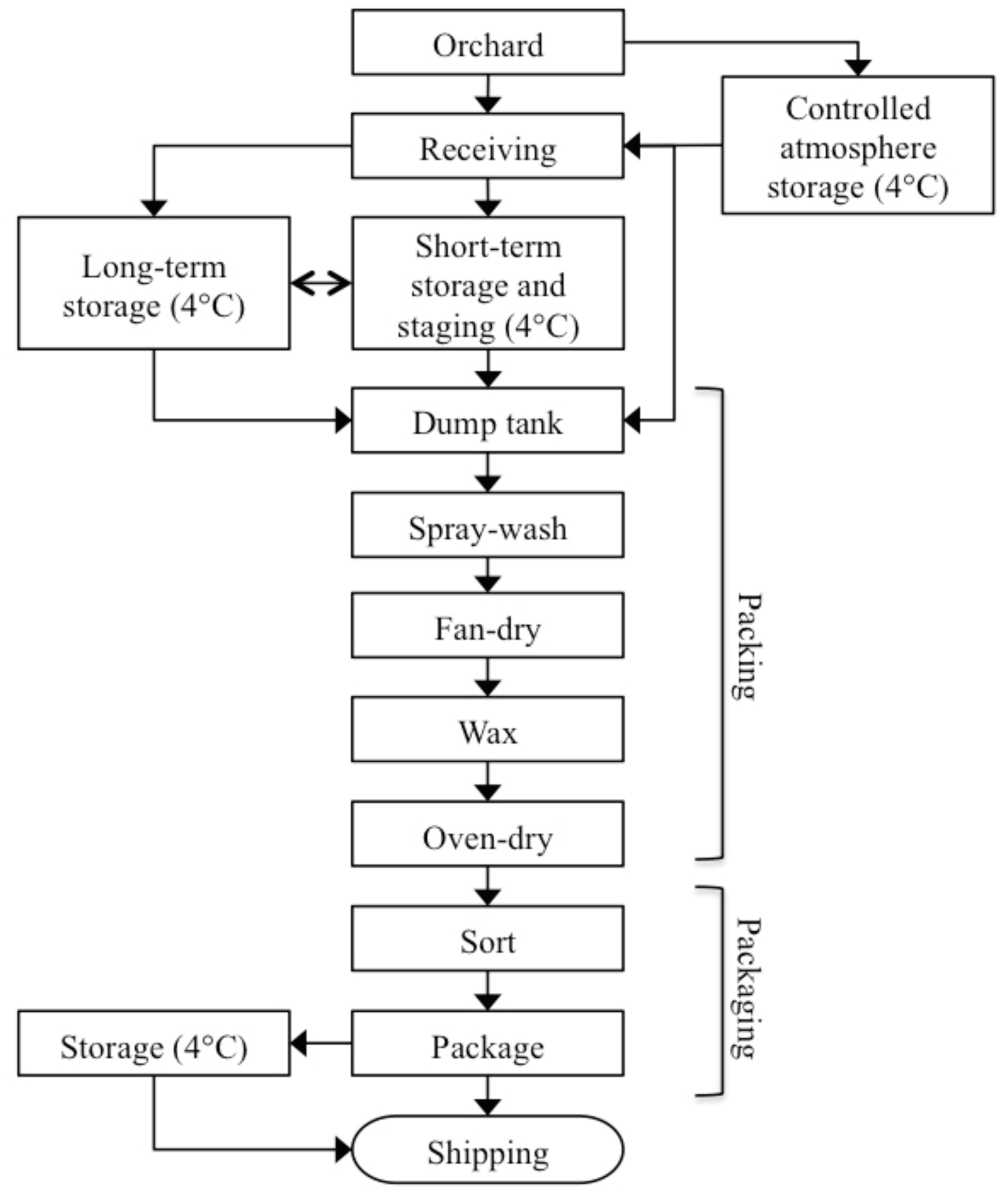
Flow diagram of tree fruit packinghouse process.

The age and condition of facilities, and self-reported food safety policies and practices employed within each of the three packinghouses were similar. The packinghouses were built between 30 and 60 years ago with additions and upgrades to equipment completed over time. Each followed the general process flow diagram in Figure 1. Bins from orchards or storage were emptied using flotation dump tanks, which were filled with hypochlorite-treated water that was continuously monitored for oxidation-reduction potential (ORP). Automatic control systems were in place at each facility to maintain ORP levels between 650 and 850 mV.

Management at each packinghouse self-reported that, at the end of each production day, they rinse food-contact and non-food-contact surfaces with water to remove visible debris. Only P2 reported using an alkaline cleaning compound following the water rinse. Each reported having used quaternary ammonium compounds (QAC) or peroxyacetic acid (PAA) antimicrobial spray solutions at varying intervals on their packing lines.

The tree fruit packing season begins in August with the arrival of peaches after which production in September shifts to early apple varieties. Depending on influx of apples arriving from the orchard and customer demand for the final product, the apples may be washed and packed immediately upon receipt (tree-run fruit), placed in refrigerated (0–4°C) rooms in the packinghouse for short-term storage and staging (0–1 months) or for longer times (1–4 months) through November as both tree-run and short- and long-term (1–3 months) stored fruit are packed. From September through November, a portion of the harvested fruit is placed into controlled atmosphere (CA) storage facilities (3–9 months). From December through March, packing activity becomes intermittent as CA stored apples are packed. Once CA stored apples are depleted in April through July, packing operations typically become inactive.

The packing line begins as forklifts unload the bins into a water flotation dump tank, where they are conveyed to a pre-sorting line for removal of misshapen and decayed apples, leaves, sticks, and foreign material. The fruit progresses across a bed of brushes, where they are spray-washed with potable water, fan-dried, and then coated with shellac or wax based formulations. The coated fruit move through a drying oven at 44–54°C for several seconds to set the wax and then into a packaging area, where they may be further sorted and graded. The apples are then packaged into boxes or plastic bags and placed in refrigerated storage (0–4°C) for eventual loading onto refrigerated trucks for shipping and distribution.

### Sampling Plan

Company management at each packinghouse granted full access to their facilities upon condition that only non-food-contact surfaces would be sampled. For purposes of this study, three area groups within the packinghouses were designated: cold storage, packing line, and packaging line (Table 1). Cold storage sites included areas within the packinghouses, where apple bins delivered from orchards were temporarily (<1 month) held or staged under refrigerated conditions before the fruit (tree-run) was moved to the packing line. As deliveries increased through the fall, bins were also placed in longer-term cold storage (1–3 months) for packing in late fall or winter as needed. Although each of the packinghouses utilized controlled atmosphere (CA) storage facilities, they were not included in this study because they were not accessible throughout the packing season. The packing line included the area, where bins were emptied by forklifts into flotation dump tanks, conveyed to an inspection line, and moved by brush rollers to overhead spray washing, fan drying, spray wax application, and oven drying. Within the packing line area, an additional sub-area was designated, where cross-contamination risks between the packing line and other areas were considered high. This sub-area included high traffic floors, catwalks, and drains, where there was a convergence of rinse water and product debris. Forklifts that crossed product area boundaries were also included in this sub-area. Within the packaging area, fruit was further inspected, sorted by size, and packaged into boxes or plastic bags.

**Table 1:** Experimental plan and description of non-food-contact sample sites over 2 years within three tree fruit packinghouses (P1, P2, and P3).

Area	No. of samples	Description of sampling sites
Cold storage	11	Floors, floor cracks and seams, and foot of fruit storage bins
Packing line	25	Bin loading/unloading equipment, top rim of dump tank, cull bins, equipment over brush bed (spray bars, apple flow partitioning dividers, and structural supports), and equipment under brush bed (drip pans, drip pan drainage funnels, brush scraper bars, structural supports, and floors) Potential cross-contamination sites: high traffic floors, catwalks, drains, and forklifts
Packaging line	4	Sorting/grading equipment, packaging equipment, and floors
Total	40	

Although samples were taken in all production areas, site selection was biased toward sites that were frequently wet, where high levels of product debris accumulated during peak packing operations (UFPA, 2018), and where preliminary sampling revealed areas at a higher risk for recurring *Listeria* contamination such as those adjacent to apple conveying and handling areas.

Each of the three packinghouses (P1, P2, and P3) was sampled twice within each of three seasonal intervals, designated as Fall (F), Winter (W), and Spring (S), which approximately bracketed anticipated active, intermittent, and inactive seasonal activity levels, respectively. Sampling was conducted over two successive years (Y1 and Y2). Actual sampling dates were chosen by taking into consideration packinghouse accessibility and time required for laboratory analysis.

### Sample Collection

Non-food-contact-surface environmental sites were sampled using sterile sponges pre-moistened with 10 ml of Dey-Engley (D/E) neutralizing buffer (Product number HS10DE2G, 3MÔ Microbiology, St. Paul, MN, United States). Floors, catwalks, drip plans, forklifts, and dump tank exteriors were sampled over an approximately 30 cm by 30 cm area by swabbing vertically five times using one side of the sponge and then horizontally five times on the reverse side of the sponge. Floor sites with noticeable cracks and seams were sampled in the same way with the addition of running both long narrow edges of the sponge along the interior of the crack or seam. Spray bars and plastic product flow partitioning surfaces between packing line sub-areas; and scraper bars and structural supports below the line were representatively sampled by sponge swabbing over a length of the accessible surface. Sterile gloves were used and changed between sampling individual sites. All samples were taken at least 3 h after start of operations and were kept on ice packs in an insulated cooler for transport on the same day to the Penn State Department of Food Science. Samples were held overnight in the laboratory at 4°C until enrichment the following day.

### Detection and Isolation of *L. monocytogenes*

Samples were enriched using standard FDA methods (Hitchins et al., 2017) with slight modifications. Sponges in the sample bags were hand massaged for 10 s after which 90 ml of sterile Buffered *Listeria* Enrichment Broth (BLEB; Oxoid LTD., Basingstoke, Hamsphire, England) was added and the sample bags were re-massaged for 10 s followed by incubation for 4 h at 30°C. After the initial incubation, 400 ml of *Listeria* Selective Enrichment Supplement (SR0149A, Thermo Fisher Scientific, Lenexa, KS, United States) was added to each sample, the bags were massaged again for 10 s, and then incubated for an additional 44 h at 30°C. The enriched samples were streaked (10 ml) onto Agar *Listeria* Ottaviani and Agosti (ALOA) and RAPID’ *L. monocytogenes* (RLM; BioRad, Hercules, CA, United States) media and then incubated for 24–48 h at 37°C. Bacterial growth was assessed after 24 h and 48 h for observation of typical presumptive *L. monocytogenes* colonies with 1–3 mm diameter blue-green colonies surrounded by an opaque white halo on ALOA and 1–3 mm diameter smooth, convex, blue-green colonies on a red or yellow background on RLM (Hitchins et al., 2017). Up to two isolated presumptive positive colonies from chromogenic medium plates were streaked onto separate trypticase soy agar plates with 0.6% yeast extract (TSAYE; BD, Sparks, MD, United States) and incubated for 48 h at 30°C (Hitchins et al., 2017). One isolated colony from each TSAYE plate was inoculated into separate sterile tubes of trypticase soy broth with 0.6% yeast extract (TSBYE; BD, Sparks, MD, United States) and incubated for 24 h at 30°C for DNA extraction the following day.

### DNA Extraction and Multiplex PCR Confirmation of *L. monocytogenes*

Prior to PCR analysis, DNA was extracted from the TSBYE isolates using the method of Queipo-Ortuño et al. (2008) with slight modifications. A total of 2 ml of overnight culture was centrifuged at 14,000 *g* for 15 min. The supernatant liquid was decanted and cells within the pellet were resuspended in 1 ml RNase free water for another centrifugation at 14,000 *g* for 10 min. After decanting, the cells were resuspended in 50 ml of RNase free water, lysed by boiling for 10 min at 95°C, and then cooled for 5 min. at 4°C. The tubes were centrifuged again at 14,000 *g* for 10 min to remove cell debris and the supernatant containing the DNA was transferred to another sterile tube for re-centrifugation at 14,000 *g* for 1 min. The tubes were stored at −20°C for no more than a month until PCR analysis was performed.

Presumptive *L. monocytogenes* isolates were confirmed with Qiagen Multiplex PCR kits (Qiagen Inc., Germantown, MD, United States) using *iap* and *lmo2234* primers specific for *Listeria* spp. and *L. monocytogenes*, respectively, as described by Chen and Knabel (2007). PCR products were separated on a 2% agarose gel *via* gel electrophoresis (BioRad T100 Thermal Cycler, BioRad, Hercules, CA, United States) at 120 V for 40 min in 0.5x Tris-borate-EDTA running buffer, post stained in a 15 mg/ml ethidium bromide solution for 25 min, and de-stained in distilled water for 20 min. PCR products were visualized at 302 nm on a UV transilluminator (EC3 310 Imagining System UVP, Upland, CA, United States) using VisionWorks LS image acquisition software (UPV, Upland, CA, United States). Samples with at least one PCR confirmed *L. monocytogenes* isolate were indicated as positive for *L. monocytogenes*. Single colonies of PCR confirmed *L. monocytogenes* were transferred from TSAYE plates to TSBYE, incubated overnight at 30°C, and stored at −80°C in 20% glycerol as stock cultures.

### Statistical Analysis

Chi-square analysis was used to test for significance differences (*α* = 0.05) in the prevalence of *L. monocytogenes* between sampling year, packinghouses, and season. Fisher’s exact test with Bonferroni correction (*α* = 0.0168) was used to analyze differences in the prevalence of *L. monocytogenes* between the three packinghouses and between the three seasons. All statistical analysis was completed using MiniTab 18 (State College, PA, United States). Because our standardized sampling scheme was intentionally biased toward selecting sites in the packing line area, statistical analysis was not conducted for *L. monocytogenes* prevalence differences between major areas and sub-areas in each of the packinghouses.

## Results

Actual sampling dates and observed packing activity levels at each packinghouse over the 2-year study are shown in Table 2. Three fewer samples than called for in the experimental plan were collected due to unavailability of a cull bin in P2 (sample #16, Table 3) that occurred once during Y1 and twice during Y2. Thus, over the 2-year study, a total of 1,437 samples were obtained from the three tree fruit packinghouses.

**Table 2:** Planned sampling intervals during Fall (F), Winter (W), and Spring (S), actual sampling dates, and observed packing activity for three packinghouses (P1, P2, and P3) over 2 years (Y1 and Y2).

	Y1			Y2		
Packing house	Sampling visits	Sampling date	Packing activity^1^	Sampling visits	Sampling date	Packing activity^1^
P1	F1	09–26-16	2	F1	September 11, 2017	2
	F2	11–07-16	0	F2	October 23, 2017	2
	W1	02–06-17	0	W1	January 22, 2018	1
	W2	03–20-17	0	W2	February 26, 2018	1
	S1	05–22-17	0	S1	May 14, 2018	1
	S2	06–26-17	0	S2	July 09, 2018	1
P2	F1	09–12-16	2	F1	September 25, 2017	2
	F2	10–24-16	2	F2	November 06, 2017	2
	W1	01–23-17	1	W1	February 05, 2018	1
	W2	02–27-17	1	W2	March 12, 2018	1
	S1	05–01-17	0	S1	April 30, 2018	0
	S2	07–31-17	2	S2	June 25, 2018	0
P3	F1	09–12-16	2	F1	September 25, 2017	2
	F2	11–07-16	2	F2	November 06, 2017	2
	W1	01–23-17	1	W1	February 05, 2018	1
	W2	03–20-17	1	W2	March 12, 2018	1
	S1	05–22-17	0	S1	April 30, 2018	0
	S2	07–31-17	2	S2	June 25, 2018	0

1 0 = no activity (seasonal shutdown), 1 = Intermittently active (≥8 h/day, 1–5 days/week), 2 = Continuously active (≥8 h/day, 5–7 days/week).

**Table 3:** Average prevalence of *L. monocytogenes* for each of 40 sites sampled 12 times over 2 years in each of three packinghouses.

Area	Sub-area	Sample site description	Zone	Sample ID#	P1	P2	P3
					*Lm* prevalence (%)		
Cold storage	Short-term and staging	Floor 1/3	3	01	**33.3**	**8.3**	**8.3**
		Floor 2/3	3	02	**66.7**	**58.3**	**8.3**
		Floor 3/3	3	03	**33.3**	**25.0**	**8.3**
		Floor crack/seam 1/2	3	04	**16.7**	**0.0**	**16.7**
		Floor crack/seam 2/2	3	05	**41.7**	**16.7**	**8.3**
		Foot of fruit storage bin	3	06	**8.3**	**0.0**	**0.0**
	Long-term	Floor 1/3	3	07	**16.7**	**16.7**	**0.0**
		Floor 2/3	3	08	**16.7**	**33.3**	**0.0**
		Floor 3/3	3	09	**0.0**	**25.0**	**16.7**
		Floor crack/seam	3	10	**16.7**	**33.3**	**0.0**
		Foot of fruit storage bin	3	11	**0.0**	**8.3**	**0.0**
Packing line	Dump tank	Rim above water line 1/2	2	12	**0.0**	**0.0**	**0.0**
		Rim above water line 2/2	2	13	**0.0**	**0.0**	**0.0**
		Bin loading equipment	2	14	**0.0**	**8.3**	**8.3**
		Bin unloading equipment	2	15	**8.3**	**0.0**	**0.0**
		Interior of cull bin	3	16	**8.3**	**33.3**	**0.0**
	Spray-wash	Spray or structural bar over brushes	2	17	**0.0**	**8.3**	**0.0**
		Drip pan	3	18	**16.7**	**8.3**	**0.0**
		Scraper or structural bar under brushes	2	19	**25.0**	**0.0**	**0.0**
		Floor directly below line	3	20	**33.3**	**91.7**	**0.0**
	Fan-dry	Structural support/flow partitions above the line	2	21	**0.0**	**41.7**	**8.3**
		Drip pan	3	22	**8.3**	**66.7**	**0.0**
		Scraper bars and drip pan funnel below line	3	23	**0.0**	**91.7**	**8.3**
		Floor directly below the line	3	24	**25.0**	**100.0**	**0.0**
	Wax	Structural support/wax spray bar over brushes	2	25	**0.0**	**33.3**	**8.3**
		Flow partition dividers/wax drip area	2	26	**8.3**	**66.7**	**0.0**
		Drip pan/wax drip area	3	27	**16.7**	**100.0**	**16.7**
		Structural support/wax drip area below the line	3	28	**0.0**	**91.7**	**16.7**
		Floor directly below line	3	29	**8.3**	**100.0**	**16.7**
	Potential cross-contamination sites	Catwalks adjacent to lines 1/2	3	30	**8.3**	**58.3**	**8.3**
		Catwalks adjacent to lines 2/2	3	31	**16.7**	**41.7**	**25.0**
		Adjacent floor drains along the packing line	3	32	**33.3**	**91.7**	**0.0**
		High traffic floor adjacent to packing line 1/2	3	33	**8.3**	**33.3**	**0.0**
		High traffic floor adjacent to packing line 2/2	3	34	**0.0**	**33.3**	**0.0**
		Forklift tine	3	35	**8.3**	**0.0**	**8.3**
		Forklift wheel	3	36	**8.3**	**16.7**	**0.0**
Packaging line		Floor directly below sorting line	3	37	**0.0**	**16.7**	**0.0**
		Sorting line equipment	2	38	**0.0**	**33.3**	**0.0**
		Floor directly below pack line	3	39	**0.0**	**16.7**	**0.0**
		Line equipment	2	40	**0.0**	**8.3**	**0.0**

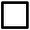
 0%, 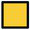 0–25%, 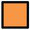 26–50%, 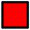
51–75%, and 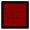 >75%.

The overall prevalence of *L. monocytogenes* within the three packinghouses over the 2-year study was 17.5% (Table 4). Prevalence values did not significantly (*p* > 0.05) differ between Y1 and Y2. Therefore, the data for each year were pooled and are presented hereafter as combined overall 2-year prevalence values. Although *L. monocytogenes* was repeatedly detected in all three packinghouses, overall prevalence values were significantly (*p* ≤ 0.05) highest in P2 with respective frequency values 2.9 and 7.4 times higher than in P1 and P3 (Table 4).

**Table 4:** Combined 2-year data for the location and occurrence of *L. monocytogenes* in each of the three packinghouses (P1, P2, and P3).

Area	Year	Samples positive for *L. monocytogenes*/Samples collected (%)			
		P1	P2	P3	Overall
**Cold storage**					
Short-term and staging^1^	Y1	10/36 (27.7)	8/36 (22.2)	2/36 (5.6)	20/108 (18.5)
	Y2	14/36 (38.9)	5/36 (13.9)	4/36 (11.1)	23/108 (21.3)
Long-term^2^	Y1	4/30 (13.3)	8/30 (26.7)	1/30 (3.3)	13/90 (14.4)
	Y2	2/30 (6.7)	6/30 (20.0)	1/30 (3.3)	9/90 (10.0)
Sub-total	Y1 + Y2	30/132 (22.8)	27/132 (20.5)	8/132 (6.1)	65/396 (16.4)
**Packing line**					
Dump tank	Y1	1/30 (3.3)	2/29 (6.9)	1/30 (3.3)	4/89 (4.5)
	Y2	1/30 (3.3)	2/28 (7.1)	0/30 (0.0)	3/88 (3.4)
Spray-wash	Y1	2/24 (8.3)	7/24 (29.1)	0/24 (0.0)	9/72 (12.5)
	Y2	7/24 (29.2)	6/24 (25.0)	0/24 (0.0)	13/72 (18.0)
Fan-dry	Y1	2/24 (8.3)	21/24 (87.5)	1/24 (4.2)	24/72 (33.3)
	Y2	2/24 (8.3)	15/24 (62.5)	1/24 (4.2)	18/72 (25.0)
Wax	Y1	2/30 (6.7)	25/30 (83.3)	6/30 (20.0)	33/90 (36.7)
	Y2	2/30 (6.7)	22/30 (73.3)	1/30 (3.3)	25/90 (27.8)
Potential cross-contamination sites	Y1	5/42 (11.9)	21/42 (50.0)	3/42 (7.1)	29/126 (23.0)
	Y2	5/42 (11.9)	12/42 (28.6)	2/42 (4.8)	19/126 (15.0)
Sub-total	Y1 + Y2	29/300 (9.7)	133/297 (44.8)	15/300 (5.0)	177/897 (19.7)
Packaging line	Y1	0/24 (0.0)	7/24 (29.1)	0/24 (0.0)	7/72 (9.7)
	Y2	0/24 (0.0)	2/24 (8.3)	0/24 (0.0)	2/72 (2.8)
Sub-total	Y1 + Y2	0/48 (0.0)	9/48 (18.8)	0/48 (0.0)	9/144 (6.3)
All samples	Y1	26/240 (10.8)	99/239 (41.4)	14/240 (5.8)	139/719 (19.3a^3^)
	Y2	33/240 (13.8)	70/238 (29.4)	9/240 (3.8)	112/718 (15.6a)
Overall	Y1 + Y2	59/480 (12.3B^3^)	169/477 (35.4C)	23/480 (4.8A)	251/1437 (17.5)

1 Staging and storage for <1 month.

2 Non-CA storage for 1–3 months.

3 Different lower or uppercase letters for each year or packinghouse, respectively, indicate significant differences (*α* = 0.05).

As anticipated in the experimental plan, peak packing activity began in F1, was more intermittent in W1, and became inactive during S1, with some exceptions. At P1 during Y1, company management shut down the packing line intended for sampling from F2 through S2 due to lower than expected fruit production. At P1 during Y2, activity remained intermittent through the Y2 season due to a late season influx of imported fruit. In P2 and P3 during Y1, packing activity during S2 became active due to an earlier than expected peach harvest.

**Figure 2:**
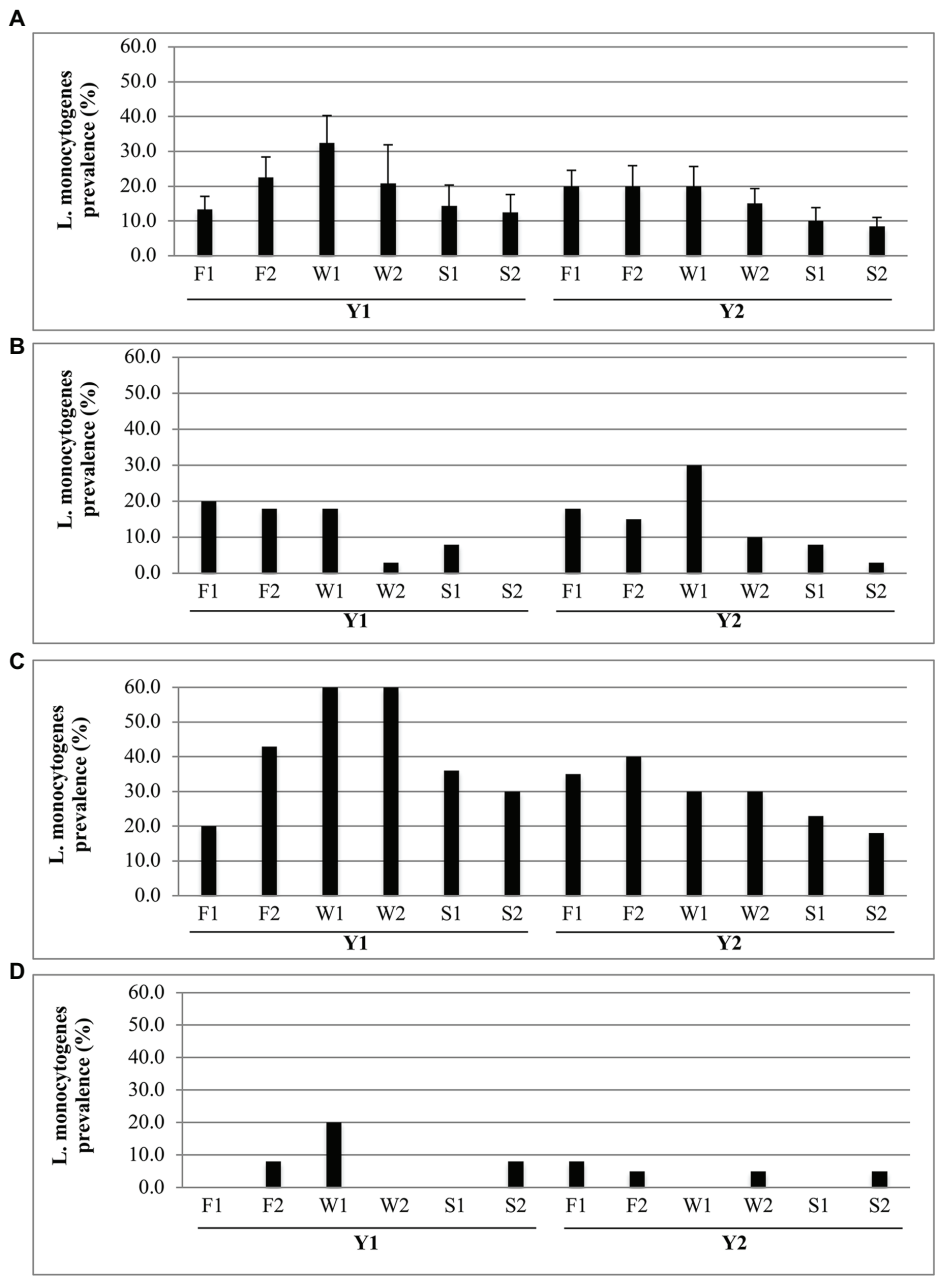
Seasonal prevalence of *Listeria monocytogenes* for each Fall (F), Winter (W), and Spring (S) sampling visit for Y1 and Y2. **(A)** Prevalence (average + SD) over all packinghouses (*N* = 3). **(B–D)** Prevalence values at each respective packinghouse (P1, P2, and P3) for each sampling visit (*N* = 40).

Significant (*p* ≤ 0.05) changes in overall *L. monocytogenes* prevalence values occurred as the packing season progressed. In Figure 2A, the combined packinghouse data show that each year levels increased from F1 through F2, peaked in early winter (W1), and then decreased through spring (S1 and S2). Although the data separated for each packinghouse show similar trends (Figures 2B–D), some exceptions are evident. In P1, despite the Y1 shut down of the packing line between F1 and F2 (Table 2), prevalence levels remained relatively constant through W1 (Figure 2B). Also, in P1, despite continued intermittent Y2 packing activity from W1 through S2 (Table 2), *L. monocytogenes* occurrence decreased during that time period (Figure 2B). In P3 during Y1 (Figure 2D), a slight increase in *L. monocytogenes* prevalence during S2 was possibly due to earlier than expected peach packing activity (Table 2). A lack of a discernable trend in P3 during Y2 was likely due to the very low overall prevalence levels for that year and location.

There were clear differences in *L. monocytogenes* prevalence values among the areas within each packinghouse. Overall prevalence values were highest in the packing line area (19.7%) followed by cold storage areas (16.4%) and the packaging area (6.3%; Table 4). Within the packing line area, *L. monocytogenes* was found in each of the five sub-areas, particularly, where spray-wash, fan-dry, and waxing activities took place. The overall packing line prevalence in P2 was 4.6 and 9.0 times greater than in P1 and P3, respectively (Table 4). In cold storage areas, *L. monocytogenes* prevalence ranking was variable although values in the short-term storage and staging sub-areas were notably higher in P1 and P2 compared to P3. Along the packaging lines, *L. monocytogenes* was detected only in P2, although at a lower rate than in packing and cold storage areas.

In Table 3, combined *L. monocytogenes* values are shown for each of 40 sites sampled 12 times over 2 years in each of the three packinghouses. Color ranking is used to display the extent to which *L. monocytogenes* was repeatedly detected at each of the sites. Overall, in P1, P2, and P3, *L. monocytogenes* was detected at least once at 25, 33, and 16 of the 40 respective sample sites. The only two sites where *L. monocytogenes* was never detected in any of the packinghouses were on the top rim of the dump tank, which was regularly subjected to splashing of chlorinated water (#12 and #13; Table 3). There are clear differences among packinghouses with respect to the number of times sample site locations were positive during more than 6 of the 12 sampling visits (>50%; Table 3). In P2, 11 of the 40 sites were positive for *L. monocytogenes* during more than half of the sampling visits; on the floor of the short-term cold storage and staging area (#2); the floor directly below the spray-wash line (#20), the drip pan (#22), the scraper bar (#23), and the floor directly below the fan-dry area (#24); the flow partition divider (#26), two wax drip sites (#27 and #28), and the floor in the wax area (#29); one of the catwalks (#30) and at a site adjacent to packing line floor drains (#32). In contrast, *L. monocytogenes* was detected at more than half of the sampling visits in P1 at only one location: the floor of the short-term cold storage and staging area (#2). In P3, *L. monocytogenes* was not detected at any site during more than half of the sampling visits. These results show that the repeated incidence of *L. monocytogenes* occurs in each of the packinghouses, but that it is highly persistent in the packing line area of P2.

## Discussion

Differences in the prevalence of *L. monocytogenes* levels in food processing and packing facilities vary depending on the type of food and process method, contamination levels on incoming ingredients and materials, degree to which facility and equipment comply with sanitary design principles, efficacy of cleaning and sanitizing procedures, and employee training policies and practices (Malley et al., 2015; UFPA, 2018). The extent to which *L. monocytogenes* was found in the three packinghouses surveyed in this study are within the wide range of prevalence values reported for retail food establishments (6.8–60%; Sauders et al., 2009; Simmons et al., 2014; Etter et al., 2017), facilities that pack or process raw and smoked fish (11.1–84.0%; Dauphin et al., 2001; Hoffman et al., 2003; Lappi et al., 2004a; Thimothe et al., 2004; Chen et al., 2010), meat and poultry (2.2–61.5%; Lawrence and Gilmour, 1994; Williams et al., 2011; Larivière-Gauthier et al., 2014), and dairy products (2.7–19.5%; Ho et al., 2007; Ruckerl et al., 2014). There are few studies documenting the prevalence of *L. monocytogenes* in fresh fruit and vegetable packing or processing facilities. *Listeria monocytogenes* prevalence on conveyer belts and cooler surfaces in multiple Texas cabbage packing sheds was 2.3% (*N* = 135; Prazak et al., 2002), less than 1% (*N* = 273) on fruit-contact surfaces in a peach packinghouse (Duvenage and Korsten, 2016), and 4.4% (*N* = 1,574) on floors, drains, and storage shelves in multiple fresh-cut fruit and vegetable facilities (Leong et al., 2014). In contrast, higher prevalence values of 33% (*N* = 39) were reported in a packinghouse linked to the 2011 cantaloupe listeriosis outbreak (FDA, 2011) and 18.7% (*N* = 255) in a mushroom packing and slicing facility not linked to any recalls or outbreaks (Murugesan et al., 2015). In a study of the prevalence of *Listeria* in 11 Eastern United States produce packinghouses (*N* = 1,588 samples), the combined *Listeria* spp. prevalence was 6.4% with more than half confirmed as *L. monocytogenes* (Estrada et al., 2020). In a study of seven produce handling and processing facilities in the Pacific Northwest sampled over two visits, *L. monocytogenes* was detected in five of the facilities where prevalence values ranged from 2 to 26% (Jorgensen et al., 2020).

There are fewer publicly available reports on the prevalence of *L. monocytogenes* in tree fruit packinghouses. In a survey of Australian tree fruit packinghouses conducted between 1999 and 2000, Portman et al. (2002) found that 12% of environmental samples were positive for *L. monocytogenes*, including multiple detections on food-contact conveyer rollers and brushes along the packing line. In a microbial survey of the packinghouse that supplied apples involved in the 2014 caramel apple listeriosis outbreak (CDC, 2015b), 6.4% of environmental samples (*N* = 110) were positive for *L. monocytogenes* (Angelo et al., 2017). Positive sites included food-contact fruit polishing and drying brushes, a conveyor, inside a wooden bin, and in a packing line floor drain, each found to be highly related to patient isolates. After reviewing preliminary data collected in the 1st year of multiyear study reported here, our group (Tan et al., 2019) also conducted a 1-year study to determine the composition of microbial communities in the packing line environment for each of the same three packinghouses. In addition to the microbiome data gathered, we found the same ranking of *L. monocytogenes* prevalence with 28, 100, and 41% reported in P1, P2, and P3, respectively (*N* = 39 for each facility).

These studies show that the detection of *L. monocytogenes* can be expected in a wide variety of food processing and packing environments, and it is not unrealistic to find it in tree fruit packinghouses. Comparing reported prevalence values to predict food contamination risks in individual facilities should be done with caution since the types of food produced in each facility, the number and distribution of sites chosen for sampling, and the analytical methods used varied among studies. Nevertheless, widespread detection of *L. monocytogenes* in the packinghouses survey in this study points to the need to establish preventive controls to keep pathogen contamination to a minimum.

The prominent cyclic seasonal patterns of *L. monocytogenes* prevalence (Figure 2) and packing activity (Table 2) strongly suggest that inadequately controlled water spillage and food debris are major factors for predicting recurring incidence of *L. monocytogenes* in packing environments. As fruit arrived from the orchard and packing began, water and product debris (microbial nutrients) were increasingly observed on floors and equipment surfaces; conditions that are known to support the survival and growth of *L. monocytogenes* in food processing facilities (Ferreira et al., 2014; Murugesan et al., 2015; Etter et al., 2017). As packing activity decreased in the spring (Table 2), equipment and floors were less frequently wet and *L. monocytogenes* contamination became less prevalent (Figure 2). This effect on *L. monocytogenes* prevalence is particularly apparent in P2 (Figure 2C).

The psychrotrophic nature of *L. monocytogenes* may have also contributed to the greater overall incidence of this pathogen in the winter (Figure 2A, W1). We did not monitor temperatures within the packinghouses in this study. However, it is possible that, during low-temperature seasons, *L. monocytogenes* had a competitive advantage over the abundant bacterial and fungal communities inhabiting tree fruit packinghouses (Tan et al., 2019). Thus, during winter months, overall conditions in packinghouses may have been more conducive for *L. monocytogenes* growth and survival.

The unequal distribution of *L. monocytogenes* in areas and sub-areas within each packinghouse (Tables 3 and 4) further points to the important role that uncontrolled water and debris are associated with increasing contamination risks. Packinghouse operations required large amounts of water to convey and wash apples, and in each facility, floors and equipment were visibly wet to varying degrees. In the packing line area, as apples were conveyed under the spray bars, water flowed over the fruit removing visible soils as it carried leaves, twigs, and debris down through the conveying brushes. During peak production times, overhead spray-washing activities generated aerosol droplets that were spread by the high-speed drying fans. In each packinghouse, drip pans were fitted under the brush beds to collect water and debris, where they were diverted toward drains. However, there were noticeable differences in the ability of each packinghouse pan configuration to contain and divert water and debris. Compared to P1 and P3, the floors under the packing line in P2 were more often saturated with water and covered with leaves, twigs, and fruit debris during peak operation periods. In P1 and P3, the drip pans were placed under the spray-wash, fan-dry, and wax sub-areas and had vertical sidewalls that contained and diverted most of the water and debris to the drains. However, the drip pan under the brush bed in P2 was a makeshift, corrugated flat, plastic (Y1), or metal (Y2) sheet with no sidewalls, and it did not extend into the fan-dry and wax sub-areas. This allowed substantial amounts of water and debris to flow directly onto the floor below and is the likely explanation for the especially high level of repeated *L. monocytogenes* detections at packing line floor sample sites (#20, #24, and #29) and equipment structural sites (#23, #26, #27, and #28) in P2 (Table 3). In contrast, cold storage and packaging areas were comparatively dryer than the packing line area and had lower *L. monocytogenes* prevalence values. An unexpectedly higher prevalence in cold storage floor sample #2 in P1 and P2 was probably due to inadequate physical separation of the packing line and storage areas as well as regular forklift movement between these areas. Elevated prevalence values at sites within the potential cross-contamination sub-area (Tables 3 and 4) are further evidence that uncontrolled movement of workers and equipment between packing line areas and the rest of the facility is a mechanism for transmitting widespread and persistent *L. monocytogenes* contamination.

Increases in *L. monocytogenes* contamination, during the most intense production periods, has been attributed to the failure of sanitation programs to maintain control of the processing environment (Tompkin, 2002). Studies on the impact of improved sanitation interventions have had mixed results and complete eradication continues to be a challenge (Lappi et al., 2004a,b; Murugesan et al., 2015; Dev Kumar et al., 2020). In facilities, where *L. monocytogenes* is persistent, more aggressive strategies may be necessary, such as making significant facility design changes, including upgrading legacy equipment, so that it meets sanitary design standards for eliminating niche sites, where bacteria can resist cleaning and sanitizing procedures (Etter et al., 2017).

Commercial wax coatings applied to apples have been shown to affect bacterial and fungal diversity and community composition of the fruit (Abdelfattah et al., 2020) and also to have a positive effect on the long-term survival of *L. monocytogenes* (Macarisin et al., 2019). Therefore, particular attention should be paid to wax application areas, where it is possible that residues could facilitate *L. monocytogenes* persistence on both non-food- and food-contact surfaces.

Sites, where the repeated detection of *L. monocytogenes* occurred (Table 3), could be harborage sites, where bacteria are protected from the action of cleaners and sanitizers and are able to survive and grow. However, they might also be locations that were repeatedly contaminated from one or more additional reservoirs. A limitation of this study is that only non-food-contact surfaces were sampled. However, the high prevalence of *L. monocytogenes* in the processing environment is associated with higher risks for product contamination (Thimothe et al., 2004). Difficult-to-clean conveyers and brushes, previously shown by Portman et al. (2002) and Angelo et al. (2017) to be susceptible to *L. monocytogenes* contamination, may be food-contact harborage sites in the packinghouses that participated in this study. Tree fruit packinghouse operators can use the results of this study to help them to identify critical non-food-contact areas for inclusion in their environmental monitoring programs.

## Conclusion and Recommendations

The results from this 2-year study of three tree fruit packinghouse showed repeated *L. monocytogenes* contamination at multiple sites in each of the tree facilities, with especially high persistence found in P2. Prevalence changes that occurred throughout the packing season and differences found between specific areas of the packhouses strongly suggest that failure to control moisture and fruit debris creates conditions that contribute to the survival and growth of *L. monocytogenes*.

Tree fruit packinghouse facilities should assess their level of compliance with sanitation standards for minimizing environmental contamination risks including FDA mandated Current Good Manufacturing Practices (GMP; Federal Register, 2015) and produce industry guidance (UFPA, 2018). In particular, preventive control measures should be implemented to limit moisture and debris accumulation in the packing environment, especially under wet packing line brush areas. Where necessary, drip pans should be replaced or structurally modified so that they are able to confine and divert water flowing under the brushes without overflowing onto floors. Efforts should be made to minimize cross-contamination risks by eliminating worker and forklift traffic between packing, storage, and packaging areas or by installing effective barriers such as footbaths and floor sanitizer foam sprays. Cleaning and sanitizing chemicals and methods for their application should be evaluated for their efficacy in removing soils, including wax residues, and for lowering microbial levels on non-food- and food-contact surfaces.

Further research is needed to conduct case studies to investigate the effectiveness of facility-wide cleaning and sanitizing policies and procedures, the extent to which they are carried out, and the strategies for developing sanitation programs that are better able to respond to daily and seasonal changes in production activity.

## Data Availability Statement

The original contributions presented in the study are included in the article/supplementary material, further inquiries can be directed to the corresponding authors.

## Author Contributions

DM: conceived and administrated this project, methodology, and review and editing. LL: critical expertise, methodology, supervision of data collection and analysis, and writing the main manuscript text. TS: data collection, analysis, writing the main manuscript text, and prepared the figures. YC: expertise, methodology, and review and editing. All authors (TS, KP, YC, QJ, GZ, LL, and DM) provided critical input and approved the final manuscript.

### Conflict of Interest

The authors declare that the research was conducted in the absence of any commercial or financial relationships that could be construed as a potential conflict of interest.

## References

[ref1] Abdelfattah A. Whitehead S. R. Macarisin D. Lu J. Burchard E. Freilich S.. (2020). Effect of washing, waxing, and low-temperature storage on the postharvest microbiome of apple. Microorganisms 8:944. 10.3390/microorganisms8060944, PMID: 32585961 PMC7356622

[ref2] Angelo K. M. Conrad A. R. Saupe A. Dragoo H. West N. Sorenson A.. (2017). Multistate outbreak of *Listeria monocytogenes* infections linked to whole apples used in commercially produced, prepackaged caramel apples: United States, 2014–2015. Epidemiol. Infect. 145, 848–856. 10.1017/S0950268816003083, PMID: 28065170 PMC6542465

[ref3] Buchanan R. L. Gorris L. G. M. Hayman M. M. Jackson T. C. Whiting R. C. (2017). A review of *Listeria monocytogenes*: an update on outbreaks, virulence, dose-response, ecology, and risk assessments. Food Control 75, 1–13. 10.1016/j.foodcont.2016.12.016

[ref4] Carpentier M. Cerf O. (2011). Review—persistence of *Listeria monocytogenes* in food industry equipment and premises. Int. J. Food Microbiol. 145, 1–8. 10.1016/j.ijfoodmicro.2011.01.005, PMID: 21276634

[ref5] CDC (2015a). Wholesome Soy Products, Inc. Sprouts and Investigation of Human Listeriosis Cases (Final Update). Centers for Disease Control and Prevention January 27, 2015. Available at: https://www.cdc.gov/listeria/outbreaks/bean-sprouts-11-14/index.html (Accessed August 22, 2019).

[ref6] CDC (2015b). Center for Disease Control and Prevention. Multistate Outbreak of Listeriosis Linked to Commercially Produced, Prepackaged Caramel Apples Made From Bidart Bros. Apples (Final update). February 12, 2015. Available at: https://www.cdc.gov/listeria/outbreaks/caramel-apples-12-14/index.html (Accessed August 22, 2019).

[ref7] CDPH (2012). California Department of Public Health. ERU Activity Summary Report (Gills Onions). Available at: https://www.cdph.ca.gov/Programs/CEH/DFDCS/CDPH%20Document%20Library/FDB/FoodSafetyProgram/EnvInvReports/fdbEIRGO2012.pdf (Accessed August 22, 2019).

[ref8] CFIA (2015). Food Recall Warning—Sliced Apples and Products Containing Sliced Apples Recalled Due to *Listeria monocytogenes*. Canadian Food Inspection Agency. April 29, 2015. Available at: http://www.inspection.gc.ca/about-the-cfia/newsroom/food-recall-warnings/complete-listing/20150429b/eng/1430375161334/1430375167258 (Accessed August 22, 2019).

[ref9] Chapin T. K. Nightingale K. K. Woroboro R. W. Wiedmann M. Strawn L. K. (2014). Geographical and meteorological factors associated with isolation of *Listeria* species in New York state produce production and natural environments. J. Food Prot. 77, 1919–1928. 10.4315/0362-028X.JFP-14-132, PMID: 25364926

[ref10] Chen Y. Burall L. S. Luo Y. Timme R. Melka D. Muruvanda T.. (2016a). *Listeria monocytogenes* in stone fruits linked to a multistate outbreak: enumeration of cells and whole-genome sequencing. Appl. Environ. Microbiol. 82, 7030–7040. 10.1128/AEM.01486-16, PMID: 27694232 PMC5118914

[ref11] Chen Y. Knabel S. J. (2007). Multiplex PCR for simultaneous detection of bacteria of the genus *Listeria*, *Listeria monocytogenes*, and major serotypes and epidemic clones of *L. monocytogenes*. Appl. Environ. Microbiol. 73, 6299–6304. 10.1128/AEM.00961-07, PMID: 17693562 PMC2075000

[ref12] Chen B. Pyla R. Kim T. Silva J. L. (2010). Incidence and persistence of *Listeria monocytogenes* in the catfish processing environment and fresh fillets. J. Food Prot. 73, 1641–1650. 10.4315/0362-028X-73.9.1641, PMID: 20828470

[ref13] Chen B. Y. Wang C. Y. Wang C. L. Fan Y. C. Weng I. T. Chou C. H. (2016b). Prevalence and persistence of *Listeria monocytogenes* in ready-to-eat tilapia sashimi processing plants. J. Food Prot. 79, 1898–1903. 10.4315/0362-028X.JFP-16-149, PMID: 28221901

[ref14] Dauphin G. Ragimbeau C. Malle P. (2001). Use of PFGE typing for tracing contamination with *Listeria monocytogenes* in three cold-smoked salmon processing plants. Int. J. Food Microbiol. 64, 51–61. 10.1016/S0168-1605(00)00442-6 11252511

[ref15] Dev Kumar G. Mis Solval K. Mishra A. Macarisin D. (2020). Antimicrobial efficacy of pelargonic acid micelles against *Salmonella* varies by surfactant, serotype and stress response. Sci. Rep. 10:10287. 10.1038/s41598-020-67223-y, PMID: 32581319 PMC7314784

[ref16] Donovan S. (2015). Listeriosis: a rare but deadly disease. Clin. Microbiol. Newsl. 37, 135–140. 10.1016/j.clinmicnews.2015.08.001

[ref17] Duvenage S. Korsten L. (2016). Effect of temperature and nutrient concentration on survival of foodborne pathogens in deciduous fruit processing environments for effective hygiene management. J. Food Prot. 79, 1959–1964. 10.4315/0362-028X.JFP-16-050, PMID: 28221909

[ref18] Estrada E. M. Hamilton A. M. Sullivan G. B. Wiedmann M. Critzer F. J. Strawn L. K. (2020). Prevalence, persistence, and diversity of *Listeria monocytogenes* and *Listeria* species in produce packinghouses in three U.S. states. J. Food Prot. 83, 277–286. 10.4315/0362-028X.JFP-19-411 31961227

[ref19] Etter A. Hammons J. Roof S. R. Simmons S. C. Tongyu W. (2017). Enhanced sanitation standard operating procedures have limited impact on *Listeria monocytogenes* prevalence in retail delis. J. Food Prot. 80, 1903–1912. 10.4315/0362-028X.JFP-17-112 29053419

[ref20] FDA (2011). Environmental Assessment: Factors Potentially Contributing to the Contamination of Fresh Whole Cantaloupe Implicated in a Multi-State Outbreak of Listeriosis. October 19, 2011. Available at: http://wayback.archive-it.org/7993/20171114022308/https:/www.fda.gov/Food/RecallsOutbreaksEmergencies/Outbreaks/ucm276247.htm (Accessed August 22, 2019).

[ref21] FDA (2014). Del Monte Fresh Produce N.A., Inc. Amends Voluntary Recall of Fresh Cut Fruit Containing Gala Red Apple in a Few States in North East Us Because of Possible Health Risk. United States Food and Drug Administration. December 17, 2014. Available at: https://wayback.archive-it.org/7993/20170406100302/https://www.fda.gov/Safety/Recalls/ArchiveRecalls/2013/ucm426419.htm (Accessed August 22, 2019).

[ref22] FDA (2015). Del Monte Fresh Produce N.A. Inc., Recalls Limited Quantity of Fresh Apples Due To Possible Health Risk. United States Food and Drug Administration. October 14, 2015. Available at: https://wayback.archive-it.org/7993/20170406093916/https://www.fda.gov/Safety/Recalls/ArchiveRecalls/2015/ucm467078.htm (Accessed August 22, 2019).

[ref23] FDA (2016a). Fresh From Texas Recalls Apple Product Because of Possible Health Risk. U.S. Food and Drug Administration. April 5, 2016. Available at: https://wayback.archive-it.org/7993/20170723003846/https://www.fda.gov/Safety/Recalls/ucm494345.htm (Accessed August 22, 2019).

[ref24] FDA (2016b). FDA Investigated Multistate Outbreak of Listeria in Dole Leafy Greens Products Produced in the Dole Facility in Springfield, Ohio. U.S. Food and Drug Administration. March 31, 2016. Available at: https://www.fda.gov/food/outbreaks-foodborne-illness/fda-investigated-multistate-outbreak-listeria-dole-leafy-greens-products-produced-dole-facility (Accessed August 22, 2019).

[ref25] FDA (2016c). Country Fresh Recalls Product Because of Possible Health Risk. U.S. Food and Drug Administration. August 26, 2016. Available at: http://wayback.archive-it.org/7993/20180425031746/ https://www.fda.gov/Safety/Recalls/ucm518335.htm (Accessed August 22, 2019).

[ref26] FDA (2017a). Jack Brown Produce, Inc. Recalls Gala, Fuji, Honeycrisp and Golden Delicious Apples Due to Possible Health Risk. U.S. Food and Drug Administration. December 19, 2017. Available at: https://www.fda.gov/Safety/Recalls/ucm589722.htm (Accessed August 22, 2019).

[ref27] FDA (2017b). Fresh Pak Inc. Recalls Lot Specific Sliced Apple Products Because of Possible Health Risk. U.S. Food and Drug Administration. December 22, 2017. Available at: https://www.fda.gov/safety/recalls/ucm590372.htm (Accessed August 22, 2019).

[ref28] FDA (2017c). Control of *Listeria monocytogenes* in Ready-to-Eat Foods: Guidance for Industry-Draft Guidance. U.S Food and Drug Administration. Center for Food Safety and Applied Nutrition. January 2017. Available at: https://www.fda.gov/media/102633/download (Accessed August 22, 2019).

[ref29] FDA (2019a). Recalls, Market Withdrawals, and Safety Alerts—Jac. Vandenberg, Inc. Recalls Fresh Peaches, Fresh Nectarines and Fresh Plums Because They May Be Contaminated With *Listeria monocytogenes*. U.S Food and Drug Administration. Center for Food Safety and Applied Nutrition Office of Regulatory Affairs. February 1, 2019. Available at: https://www.fda.gov/safety/recalls-market-withdrawals-safety-alerts/jac-vandenberg-inc-recalls-fresh-peaches-fresh-nectarines-and-fresh-plums-because-they-may-be (Accessed August 22, 2019).

[ref30] FDA (2019b). Henry Avocado Recalls Whole Avocados Because of Possible Health Risk. U.S. U.S Food and Drug Administration. Office of Regulatory Affairs. March 25, 2019. Available at: https://www.fda.gov/safety/recalls-market-withdrawals-safety-alerts/henry-avocado-recalls-whole-avocados-because-possible-health-risk (Accessed August 22, 2019).

[ref31] FDA (2020). Outbreak Investigation of *Salmonella* enteritidis: Peaches. U.S. Food and Drug Administration. Center for Food Safety and Applied Nutrition Office of Regulatory Affairs. October 16, 2020. Available at: https://www.fda.gov/food/outbreaks-foodborne-illness/outbreak-investigation-salmonella-enteritidis-peaches-august-2020?utm_medium=email&utm_source=govdelivery (Accessed October 17, 2020).

[ref32] Federal Register (2015). Current Good Manufacturing Practice and Hazard Analysis and Risk-Based Preventive Controls for Human Food. Washington, DC: Food and Drug Administration. September 17, 2015. Available at: https://www.federalregister.gov/documents/2015/09/17/2015-21920/current-good-manufacturing-practice-hazard-analysis-and-risk-based-preventive-controls-for-human (Accessed August 31, 2020).

[ref33] Ferreira V. Wiedmann M. Teixeira P. Stasiewicz M. J. (2014). *Listeria monocytogenes* persistence in food-associated environments: epidemiology, strain characteristics, and implications for public health. J. Food Prot. 77, 150–170. 10.4315/0362-028X.JFP-13-150, PMID: 24406014

[ref34] Garner D. Kathariou S. (2016). Fresh produce–associated listeriosis outbreaks, sources of concern, teachable moments, and insights. J. Food Prot. 79, 337–344. 10.4315/0362-028X.JFP-15-387, PMID: 26818997

[ref35] Gaul L. K. Farag N. H. Shim T. Kingsley M. A. Silk B. J. Hyytia-Trees E. (2013). Hospital-acquired listeriosis outbreak caused by contaminated diced celery—Texas, 2010. Clin. Infect. Dis. 56, 20–26. 10.1093/cid/cis817, PMID: 22997210

[ref36] Glass K. A. Golden M. C. Wandless B. J. Bedale W. Czuprynski C. (2003). Growth of *Listeria monocytogenes* within a caramel-coated apple microenvironment. MBio 6, 2–6. 10.1128/mBio.01232-15, PMID: 26463161 PMC4620460

[ref37] Gombas D. E. Chen Y. Clavero R. S. Scott V. N. (2003). Survey of *Listeria monocytogenes* in ready-to-eat foods. J. Food Prot. 66, 559–569. 10.4315/0362-028X-66.4.559, PMID: 12696677

[ref38] Hitchins A. Jinneman K. Chen Y. (2017). Bacteriological Analytical Manual Chapter 10: Detection and Enumeration of *Listeria monocytogenes* in Foods. Silver Spring, MD, US Food and Drug Administration. Available at: http://www.fda.gov/Food/FoodScienceResearch/LaboratoryMethods/ucm071400.htm (Accessed August 30, 2019).

[ref39] Ho A. J. Lappi V. R. Wiedmann M. (2007). Longitudinal monitoring of *Listeria monocytogenes* contamination patterns in a farmstead dairy processing facility. J. Dairy Sci. 90, 2517–2524. 10.3168/jds.2006-392, PMID: 17430956

[ref40] Hoffman A. D. Gall K. L. Norton D. M. Wiedmann M. (2003). *Listeria monocytogenes* contamination patterns for the smoked fish processing environment and for raw fish. J. Food Prot. 66, 52–60. 10.4315/0362-028X-66.1.52, PMID: 12540181

[ref41] Jackson B. R. Salter M. Tarr C. Conrad A. Harvey E. Steinbock L.. (2015). Notes from the field: listeriosis associated with stone fruit—United States. Morb. Mortal. Wkly Rep. 64, 282–283. PMID: 25789745 PMC4584806

[ref42] Jorgensen J. Waite-Cusic J. Kovacevic J. (2020). Prevalence of *Listeria* spp. in produce handling and processing facilities in the Pacific northwest. Food Microbiol. 90:103468. 10.1016/j.fm.2020.103468, PMID: 32336359

[ref43] Kim S. W. Haendiges J. Keller E. N. Myers R. Kim A. Lombard J. E.. (2018). Genetic diversity and virulence profiles of *Listeria monocytogenes* recovered from bulk tank milk, milk filters, and milking equipment from dairies in the United States (2002 to 2014). PLoS One 13:e0197053. 10.1371/journal.pone.0197053, PMID: 29742151 PMC5942804

[ref44] Lappi V. R. Thimothe J. Nightingale K. K. Gall K. Scott V. N. Wiedmann M. (2004a). Longitudinal studies on *Listeria* in smoked fish plants: impact of intervention strategies on contamination patterns. J. Food Prot. 67, 2500–2514. 10.4315/0362-028x-67.11.2500, PMID: 15553634

[ref45] Lappi V. R. Thimothe J. Walker J. Bell B. Gall K. Moody M. W.. (2004b). Impact of intervention strategies on *Listeria* contamination patterns in crawfish processing plants: a longitudinal study. J. Food Prot. 67, 1163–1169. 10.4315/0362-028x-67.6.1163, PMID: 15222544

[ref46] Larivière-Gauthier G. Letellier A. Kérouanton A. Bekal S. Quessy S. Fournaise S.. (2014). Analysis of *Listeria monocytogenes* strain distribution in a pork slaughter and cutting plant in the province of Quebec. J. Food Prot. 77, 2121–2128. 10.4315/0362-028X.JFP-14-192, PMID: 25474060

[ref47] Lawrence L. M. Gilmour A. (1994). Incidence of *Listeria* spp. and *Listeria monocytogenes* in a poultry processing environment and in poultry products and their rapid confirmation by multiplex PCR. Appl. Environ. Microbiol. 60, 4600–4604. PMID: 7811096 10.1128/aem.60.12.4600-4604.1994 PMC202027

[ref48] Leong D. Alvarez-Ordonez A. Jordan K. (2014). Monitoring occurrence and persistence of *Listeria monocytogenes* in foods and food processing environments in the Republic of Ireland. Front. Microbiol. 5:436. 10.3389/fmicb.2014.00436, PMID: 25191314 PMC4138519

[ref49] Macarisin D. Sheth I. Hur M. Wooten A. Kwon H. J. Gao Z.. (2019). Survival of outbreak, food, and environmental strains of *Listeria monocytogenes* on whole apples as affected by cultivar and wax coating. Sci. Rep. 9:12170. 10.1038/s41598-019-48597-0, PMID: 31434982 PMC6704171

[ref50] Malley T. J. V. Butts J. Wiedmann M. (2015). Seek and destroy process: *Listeria monocytogenes* process controls in the ready-to-eat meat and poultry industry. J. Food Prot. 780, 436–445. 10.4315/0362-028X.JFP-13-507, PMID: 25710164

[ref51] Marus J. R. Bidol S. Altman S. M. Oni O. Parker-Strobe N. Otto M.. (2019). Notes from the field: outbreak of listeriosis likely associated with prepackaged caramel apples—United States, 2017. Morb. Mortal. Wkly Rep. 68, 76–77. 10.15585/mmwr.mm6803a5, PMID: 30677010

[ref52] Murugesan L. Kucerova Z. Knabel S. J. LaBorde L. F. (2015). Predominance and distribution of a persistent *Listeria monocytogenes* clone in a commercial fresh mushroom processing environment. J. Food Prot. 78:1988. 10.4315/0362-028X.JFP-15-195, PMID: 26555522

[ref53] Portman T. Frankish E. McAlpine G. (2002). Guidelines for the Management of Microbial Food Safety in Fruit Packing Houses. Department of Agriculture and Food, Western Australia, Perth. Bulletin 4567. Available at: http://researchlibrary.agric.wa.gov.au/bulletins/69 (Accessed August 22, 2019).

[ref54] Prazak A. M. Murano E. A. Mercado I. Acuff G. R. (2002). Prevalence of *Listeria monocytogenes* during production and postharvest processing of cabbage. J. Food Prot. 65, 1728–1734. 10.4315/0362-028X-65.11.1728, PMID: 12430693

[ref55] Queipo-Ortuño M. I. De Dios Colmenero J. Macias M. Bravo M. J. Morata P. (2008). Preparation of bacterial DNA template by boiling and effect of immunoglobulin g as an inhibitor in real-time PCR for serum samples from patients with brucellosis. Clin. Vaccine Immunol. 15, 293–296. 10.1128/CVI.00270-07, PMID: 18077622 PMC2238042

[ref56] Rothrock M. J. Micciche A. C. Bodie A. R. Ricke S. C. (2019). *Listeria* occurrence and potential control strategies in alternative and conventional poultry processing and retail. Front. Sustain. Food Syst. 3:33. 10.3389/fsufs.2019.00033

[ref57] Ruckerl I. Muhterem-Uyar M. Muri-Klinger S. Wagner K.-H. Wagner M. Stessl B. (2014). *Listeria monocytogenes* in a cheese processing facility: learning from contamination scenarios over three years of sampling. Int. J. Food Microbiol. 189, 98–105. 10.1016/j.ijfoodmicro.2014.08.001, PMID: 25136788

[ref58] Salazar J. K. Carstens C. K. Bathija V. M. Narula S. S. Parish M. Tortorello M. L. (2016). Fate of *Listeria monocytogenes* in fresh apples and caramel apples. J. Food Prot. 79, 696–702. 10.4315/0362-028X.JFP-15-442, PMID: 27296414

[ref59] Sauders B. D. Sanchez M. D. Rice D. H. Corby J. Stich S. Fortes E. D.. (2009). Prevalence and molecular diversity of *Listeria monocytogenes* in retail establishments. J. Food Prot. 72, 2337–2349. 10.4315/0362-028X-72.11.2337, PMID: 19903398

[ref60] Scallan E. Hoekstra R. M. Angulo F. J. Tauxe R. V. Widdowson M. A. Roy S. L.. (2011). Foodborne illness acquired in the United States—major pathogens. Emerg. Infect. Dis. 17, 7–15. 10.3201/eid1701.P11101, PMID: 21192848 PMC3375761

[ref61] Self J. L. Conrad A. Stroika S. Jackson A. Whitlock L. Jackson K. A.. (2019). Multistate outbreak of listeriosis associated with packaged leafy green salads, United States and Canada, 2015–2016. Emerg. Infect. Dis. 25, 1461–1468. 10.3201/eid2508.180761, PMID: 31310227 PMC6649349

[ref62] Sheng L. Edwards K. Tsai H.-C. Hanrahan I. Zhu M. (2017). Fate of *Listeria monocytogenes* on fresh apples under different storage temperatures. Front. Microbiol. 8:1396. 10.3389/fmicb.2017.01396, PMID: 28790993 PMC5522875

[ref63] Sheth I. Li F. Hur M. Laasri A. De Jesus A. J. Kwon H. J.. (2018). Comparison of three enrichment schemes for the detection of low levels of desiccation-stressed *Listeria* spp. from select environmental surfaces. Food Control 84, 493–498. 10.1016/j.foodcont.2017.08.022

[ref64] Simmons C. Stasiewicz M. J. Wright E. Warchocki S. Roof S. Kause J. R.. (2014). *Listeria monocytogenes* and *Listeria* spp. contamination patterns in retail delicatessen establishments in three U.S. states. J. Food Prot. 77, 1929–1939. 10.4315/0362-028X.JFP-14-183, PMID: 25364927

[ref65] Strawn L. K. Fortes E. D. Bihn E. A. Nightingale K. K. Gröhn Y. T. Worobo R. W.. (2013a). Landscape and meteorological factors affecting prevalence of three food-borne pathogens in fruit and vegetable farms. Appl. Environ. Microbiol. 79, 588–600. 10.1128/AEM.02491-12, PMID: 23144137 PMC3553790

[ref66] Strawn L. K. Gröhn Y. T. Warchocki S. Worobo R. W. Bihn E. A. Wiedmann M. (2013b). Risk factors associated with *Salmonella* and *Listeria monocytogenes* contamination of produce fields. Appl. Environ. Microbiol. 79, 7618–7627. 10.1128/AEM.02831-13, PMID: 24077713 PMC3837806

[ref67] Swaminathan B. Gerner-Smidt P. (2007). The epidemiology of human listeriosis. Microbes Infect. 9, 1236–1243. 10.1016/j.micinf.2007.05.011, PMID: 17720602

[ref68] Tan X. Chung T. Chen Y. Macarisin D. LaBorde L. Kovac J. (2019). The occurrence of *Listeria monocytogenes* is associated with built environment microbiota in three tree fruit processing facilities. Microbiome 7:115. 10.1186/s40168-019-0726-2, PMID: 31431193 PMC6702733

[ref69] Thimothe J. Nightingale K. K. Gall K. Scott V. N. Wiedmann M. (2004). Tracking of *Listeria monocytogenes* in smoked fish processing plants. J. Food Prot. 67, 328–341. 10.4315/0362-028X-67.2.328, PMID: 14968966

[ref70] Tompkin R. (2002). Control of *Listeria monocytogenes* in the food-processing environment. J. Food Prot. 65, 709–725. 10.4315/0362-028X-65.4.709, PMID: 11952224

[ref71] UFPA (2018). Guidance on Environmental Monitoring and Control of *Listeria* for the Fresh Produce Industry. 2nd Ed. United Fresh Produce Association. Available at: https://www.unitedfresh.org/guidance-on-environmental-monitoring-and-control-of-listeria-for-the-fresh-produce-industry-2nd-ed/ (Accessed August 22, 2019).

[ref72] Williams S. K. Roof S. Boyle E. A. Burson D. Thippareddi H. Geornaras I.. (2011). Molecular ecology of *Listeria monocytogenes* and other *Listeria* species in small and very small ready-to-eat meat processing plants. J. Food Prot. 74, 63–77. 10.4315/0362-028X.JFP-10-097, PMID: 21219764

[ref73] Zhu Q. Gooneratne R. Hussain M. A. (2017). *Listeria monocytogenes* in fresh produce: outbreaks, prevalence and contamination levels. Foods 6:21. 10.3390/foods6030021, PMID: 28282938 PMC5368540

[ref74] Zoellner C. Ceres K. Ghezzi-Kopel K. Weidmann M. Ivanek R. (2018). Design elements of *Listeria* environmental monitoring programs in food processing facilities: a scoping review of research and guidance materials. Compr. Rev. Food Sci. Food Saf. 17, 1156–1171. 10.1111/1541-4337.12366, PMID: 33350161

